# Editorial: Biomarkers in migraine beyond diagnosis

**DOI:** 10.3389/fneur.2022.1096531

**Published:** 2022-12-09

**Authors:** Zhiye Chen, Xiaoyan Chen, Yan Fan

**Affiliations:** ^1^Department of Radiology, Hainan Hospital of Chinese PLA General Hospital, Sanya, China; ^2^Department of Neurology, First Medical Center of Chinese PLA General Hospital, Beijing, China; ^3^Department of Psychology and Neurosciences, Leibniz Research Centre for Working Environment and Human Factors, TU Dortmund (IfADo), Dortmund, Germany

**Keywords:** migraine, headache, biomarker, diagnosis, treatment

Migraine is a disabling type of primary headache that directly affects more than one billion people worldwide. Recent studies have provided important new insights into its genetic causes, anatomical and physiological features, and pharmacological mechanisms. In current clinical practice, migraine was diagnosed according to the International Classification of Headache Disorders (ICHD-3 criteria). Although the evolution of this classification system reflects an increasing understanding of the heterogeneity and variable clinical features of migraine, the diagnosis and treatment remain inadequate.

One of the main barriers to the precision diagnosis and treatment of migraine is the lack of reliable biomarkers. Biomarkers can have a wide range of clinical applications, including diagnosis, subtype classification, prognosis, and treatment effect assessment. The specific, individualized, and multi-perspective biomarkers of migraine can significantly promote the accurate diagnosis of migraine, and promote the exploration of pathophysiology and new treatment strategies for migraine.

To improve clinical decision-making for migraine, this Research Topic aimed to identify the potential biomarkers for migraine and to further investigate the association of biomarkers with diagnosis, stratification, prognosis, and therapy.

Nine articles had been finally included in this Research Topic, containing seven pieces of original research, one opinion, and one review.

Genetic, environmental, metabolic, and neuropeptides may all be involved in the pathogenesis of migraine. Some substances can be detected in serum and may thus serve as corresponding biomarkers. Four studies explored changes in serum concentrations of substances in migraine patients, respectively paying close attention to potential cation channel subfamily V member 1 (TRPV1), vasoacive intestinal peptide (VIP), and pituitary adenylate cyclase-activating polypeptide (PACAP) (Togha et al.), urate (Hong et al.), immunoglobulin G Glycosylation (Xu et al.), and Calcitonin gene-related peptide (CGRP) (Frank et al.).

Besides serum studies, previous neuroimaging studies have explored structural and functional changes in the brain of migraine patients, but few studies have explored biological markers associated with drug efficacy and predicting refractory migraine attacks. Three articles are related to diagnostic methods using neuroimaging: predicting sumatriptan treatment response in persons with migraine disease through neuroimaging (Wu et al.) and volume or diffusion abnormalities (Santoro et al.).

One study focused on the alteration of gut microbiota in migraine patients and investigated migraine combined with irritable bowel syndrome (Liu et al.).

Migraine has a certain genetic predisposition, and genes may play a role in its diagnosis. One opinion discussed the use of gene prioritization to score and rank suggestive candidate genes in migraine (Frederiksen).

Studies in recent years have proven that multi-functional neuropeptide CGRP plays a major role in the pathophysiology of migraine. The article (Kamm) on this topic reviewed the current understanding of CGRP in migraine pathophysiology and presented the possible applications of CGRP as a migraine biomarker.

In conclusion, published articles confirmed the complexity of migraine pathogenesis. Therefore, objective diagnostic biomarkers and personalized treatment strategies were needed. Furthermore, the clinical evaluation of patients should be comprehensive, based on large sample clinical studies and extensive evidence-based studies.

## Author contributions

All authors listed have made a substantial, direct, and intellectual contribution to the work and approved it for publication.

